# Overexpression of a Senescence-Related Gene *CpSRG1* from Wintersweet (*Chimonanthus praecox*) Promoted Growth and Flowering, and Delayed Senescence in Transgenic *Arabidopsis*

**DOI:** 10.3390/ijms232213971

**Published:** 2022-11-12

**Authors:** Yinzhu Cao, Guixiang Li, Xia Wang, Renwei Huang, Jianghui Luo, Mingyang Li, Daofeng Liu, Shunzhao Sui

**Affiliations:** Chongqing Engineering Research Center for Floriculture, Key Laboratory of Horticulture Science for Southern Mountainous Regions of Ministry of Education, College of Horticulture and Landscape Architecture, Southwest University, Chongqing 400715, China

**Keywords:** promoter, the 2OG-Fe(II) dioxygenase superfamily, transgenic *Arabidopsis*, senescence, wintersweet

## Abstract

Plant senescence is a complex process that is controlled by developmental regulation and genetic programs. A senescence-related gene *CpSRG1*, which belongs to the 2OG-Fe(II) dioxygenase superfamily, was characterized from wintersweet, and the phylogenetic relationship of CpSRG1 with homologs from other species was investigated. The expression analysis by qRT-PCR (quantitative real-time PCR) indicated that *CpSRG1* is abundant in flower organs, especially in petals and stamens, and the highest expression of *CpSRG1* was detected in stage 6 (withering period). The expression patterns of the *CpSRG1* gene were further confirmed in *CpSRG1pro::GUS* (*β-glucuronidase*) plants, and the activity of the *CpSRG1* promoter was enhanced by exogenous Eth (ethylene), SA (salicylic acid), and GA_3_ (gibberellin). Heterologous overexpression of *CpSRG1* in *Arabidopsis* promoted growth and flowering, and delayed senescence. Moreover, the survival rates were significantly higher and the root lengths were significantly longer in the transgenic lines than in the wild-type plants, both under low nitrogen stress and GA_3_ treatment. This indicated that the *CpSRG1* gene may promote the synthesis of assimilates in plants through the GA pathway, thereby improving growth and flowering, and delaying senescence in transgenic *Arabidopsis*. Our study has laid a satisfactory foundation for further analysis of senescence-related genes in wintersweet and wood plants. It also enriched our knowledge of the 2OG-Fe(II) dioxygenase superfamily, which plays a variety of important roles in plants.

## 1. Introduction

Plant senescence is a complex process that is controlled by developmental regulation and genetic programs. Senescence can also be initiated by an environmental signal [1], such as lack of minerals or water. Senescence marks a fundamental shift in plant physiology, and it ultimately leads to growth cessation or death of specific organs or entire plants. This process includes the senescence of many different tissues such as the root, stem, leaves, petals, stamens, pistils and germinating seeds, which usually involves physiolog-ical, biochemical, and molecular changes [2]. Leaf senescence manifests as the loss of photosynthesis capacity, and disintegration of chloroplasts, proteins, lipids, and nucleic acids [3,4]. The symptoms of flower senescence are the dehydration of petals, loss of floral fragrance, fading or discoloration of flower color, and curling or wilting of petals [5].

In addition to these physiological and biochemical changes, the process of senescence is also strictly controlled by multiple internal and external signals through complex molecular regulatory networks [6]. These signals originate from regulatory genes such as NAC transcription factor, zinc-finger protein, MYB transcription factors [7,8], signal transduction genes such as various protein kinases [9], and functional genes, such as defense-related genes that are also related to senescence [10].

The 2OG-Fe(II) dioxygenase superfamily is the second largest protease family in plants, and it plays a role in a variety of physiological processes, including DNA demethylation, and the biosynthesis of plant hormones and plant secondary metabolites [11,12]. In addition, it was reported that this family of genes is involved in the regulation of plant senescence. For example, the deletion mutant of the 2-oxoglutarate-dependent dioxygenase gene *F6′H1* exhibited more optimal cell membrane integrity, and higher chlorophyll content and photosynthetic efficiency in leaves as compared to the wild-type, which indicated that the *F6′H1* gene promoted leaf senescence in *Arabidopsis*. The salicylic acid 3-hydroxylase gene *AtS3H* can hydroxylate salicylic acid to 2,3-dihydroxybenzoic acid (2,3-DHBA), thereby reducing the salicylic acid content in cells to inhibit leaf senescence [13]. 

New members of the 2OG-Fe(II) dioxygenase superfamily, senescence-related genes (*SRGs*), have been subsequently found in plants. According to previous reports, SRG proteins are widely involved in plant developmental and physiological processes, and various biotic and abiotic stress responses. For example, phosphate starvation stress caused a 2.95-fold downregulation of *SRG1* expression in *Arabidopsis thaliana* [14], and drought stress induced the expression of *SRG1* [15]. In addition, low nitrogen [16] and bacterial infiltration [17] can also induce the expression of the *SRG1* gene. In tomatoes, *SRG1* is regulated by ethylene and participates in the plant defense response [18]. Moreover, the *SRG1* homologous gene is highly correlated with cell wall synthesis genes during internode elongation in maize [19]. *Citrus clementina* mutants 39B3 and 39E7 exhibited a delayed color change, and during this process, the *SRG1* gene expression level was significantly downregulated [20]. In many cases, the *SRG1* gene is used as a molecular marker for plant aging or senescence [21,22]. 

Wintersweet (*Chimonanthus praecox*) is a perennial deciduous shrub of the *Calycanthaceae* that is native to China. It flowers in winter with sweet fragrance, and is widely used as a landscape shrub, potted plant, and for woody cut flowers in China. Due to its unique flowering time, some genes related to wintersweet flower development and senescence have been identified and studied. For example, *CpAGL6*, a SEP1-like gene, caused abnormal stamens, decreased fertility and affected carpel development in transgenic *Arabidopsis* [23]. A WRKY transcription factor gene *CpWRKY71* may function in regulating flowering time and leaf senescence in transgenic *Arabidopsis* [24]. Additionally, for many plants, the research on senescence is always one of the hot issues, which related to its commercial value and market competitiveness. However, the senescence-related molecular mechanism is still unclear. 

To the best of our knowledge, the *SRGs* have not been well studied in model or non-model plants, or even in woody plants. In the current study, we isolated and characterized a senescence-related gene, *CpSRG1*, from wintersweet. The expression pattern of *CpSRG1* was investigated. The promoter sequence of *CpSRG1* was isolated, and the expression features and promoter activity were investigated using the GUS reporter gene driven by the *CpSRG1* promoter. To verify the function of *CpSRG1*, we heterologously expressed *CpSRG1* in *Arabidopsis*. In addition, the transgenic *Arabidopsis* was treated with low nitrogen and gibberellin to clarify the response of *CpSRG1*. Overall, this study has laid a satisfactory foundation for further analysis of senescence-related genes in wintersweet. It provides clues regarding the roles that the *SRG1* gene may play in woody plants through its functional characterization. Moreover, these finding also enrich our knowledge of the 2OG-Fe(II) dioxygenase superfamily, which plays a variety of important roles in plants. 

## 2. Results

### 2.1. Isolation and Characterization of CpSRG1 from Wintersweet

**Figure 1:**
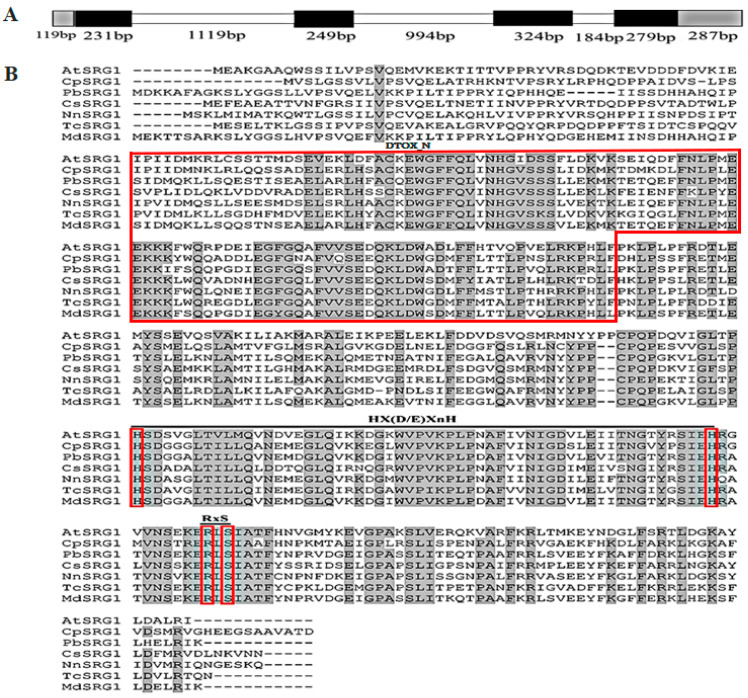
Sequence analysis of CpSRG1. (**A**) Schematic diagram of the *CpSRG1* gene. Exons are indicated by black areas, introns are indicated by white areas, and untranslated regions are indicated by gray areas. (**B**) Multiple alignment of CpSRG1 and other SRG1 proteins from different plant species. *Arabidopsis thaliana* AtSRG1 (NP_173145), *Pyrus bretschneideri* PbSRG1 (KAB2605442.1), *Citrus sinensis* CsSRG1 (XP_006477808.1), *Nelumbo nucifera* NnSRG1-LIKE (XP_010273388.1), *Theobroma cacao* TcSRG1 (EOY13557.1), and *Malus domestica* MdSRG1-LIKE (XP_028944961.1). Identical and similar amino acids are shaded in black. The conserved Hx(D/E) XnH motif, RxS motif, and DIOX_N are marked by a red box.

Based on the cDNA library of wintersweet flower, a 1489 bp cDNA of *CpSRG1* was obtained. Sequence analysis showed that *CpSRG1* contained an open reading frame (ORF) of 1083 bp, which was predicted to encode a 360 amino acid (aa) protein, with a molecular weight of 40.16 kDa and the theoretical isoelectric point of 5.96. Moreover, we isolated a 3786 bp genomic DNA fragment of *CpSRG1* that contained three introns (1119 bp, 994 bp, and 184 bp) and four exons (231 bp, 249 bp, 324 bp, and 279 bp) (Figure 1A).

**Figure 2:**
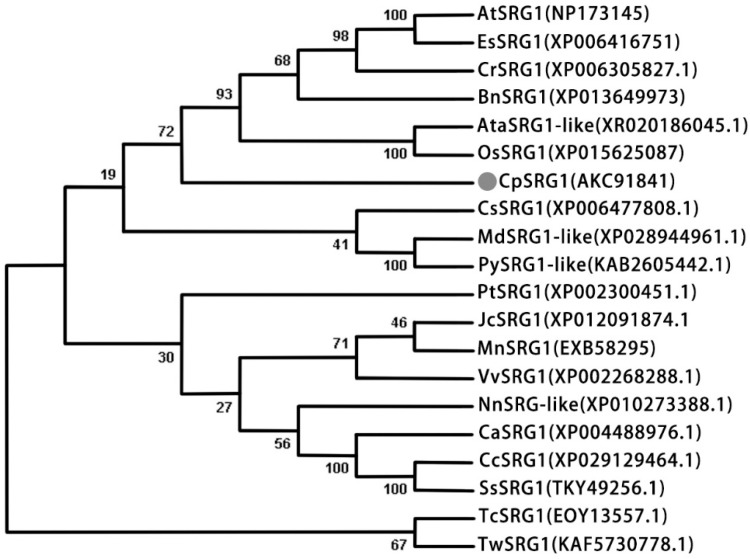
A phylogenetic tree of CpSRG1 and SRG1 proteins from other species. Phylogenetic analysis was performed by the neighbor-joining (NJ) method with 1000 bootstrap replicates using MEGA 7.0. At, *Arabidopsis thaliana;* Es, *Eutrema salsugineum*; Cr, *Capsella rubella*; Bn, *Brassica napus*; Ata, *Aegilops tauschii* subsp. *Tauschii*; Os, *Oryza sativa;* Cp, *Chimonanthus praecox;* Cs, *Citrus sinensis*; Md, *Malus domestica*; Py, *Pyrus ussuriensis* x *Pyrus communis*; Pt, *Populus trichocarpa*; Jc, *Jatropha curcas*; Mn, *Morus notabilis*; Vv, *Vitis vinifera* L.; Nn, *Nelumbo nucifera*; Ca, *Cicer arietinum*; Cc, *Cajanus cajan*; Ss, *Spatholobus suberectus*; Tc, *Theobroma cacao*; Tw, *Tripterygium wilfordii*.

BLAST analysis illustrated that CpSRG1 shares a high sequence similarity with other plant SRG1 proteins, and exhibited 61%, 60%, and 60% identity with NnSRG1 (XP_010273388.1), PbSRG1 (XP_009376721), and TcSRG1 (EOY13557.1), respectively. The multiple alignments of plant SRG1 protein sequences demonstrated that the CpSRG1 protein has a conserved domain of the 2-oxoglutarate/Fe(II)-dependent dioxygenase superfamily, a ferrous iron (II) binding residue motif (the HxDxnH motif), and the 2-oxoglutarate binding residue motif (the RxS motif). Several amino acid residues conserved in the 2-OGD superfamily were also found, including Gly74, His81, Pro209, and Gly264. The DIOX_N motif at the N-terminal region, which is highly conserved in the DOXC class of the 2-OGD superfamily, was also found in CpSRG1 (Figure 1B). The phylogenetic tree of CpSRG1 and other SRG1 homologous proteins showed that CpSRG1 was closely related to OsSRG1 and AtaSRG1 (Figure 2). 

### 2.2. The Expression Pattern of CpSRG1

**Figure 3:**
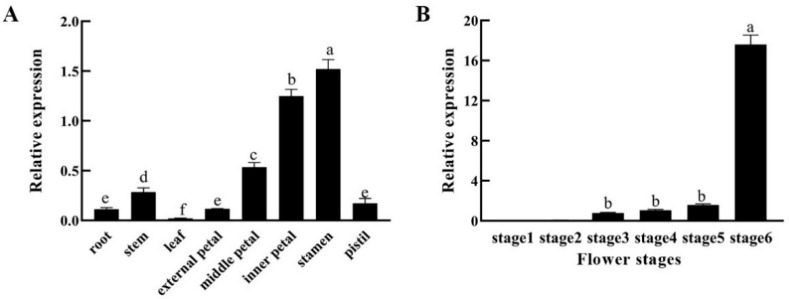
Expression patterns of *CpSRG1* in wintersweet. (**A**) Relative expression of *CpSRG1* gene in different tissues of wintersweet. (**B**) Expression of the *CpSRG1* gene at different flower stages of wintersweet. Stage 1 (sprout period); stage 2 (flower-bud period); stage 3 (petal-display period); stage 4 (initiation of bloom period); stage 5 (bloom period); stage 6 (withering period). *CpActin* and *CpTublin* were used as an internal control. Data represent the mean of three biological repeats ± SEM. Error bars indicate the standard error of the mean. Different lowercase letters (a–f) on bars indicate significant differences (*p* < 0.05).

The expression pattern of *CpSRG1* in different tissues of wintersweet was analyzed by quantitative real-time PCR. The results showed that the expression of *CpSRG1* was abundant in flower organs, especially in petals and stamens. In vegetative organs, the expression level of *CpSRG1* was higher in the stem than in the root or leaf (Figure 3A). Moreover, the expression pattern of the *CpSRG1* gene at different flower stages was also examined. The highest *CpSRG1* expression was detected in stage 6 (withering period), followed by stage 5 (bloom period), stage 4 (initiation of bloom period), and stage 3 (petal-display period). The expression of *CpSRG1* was nearly undetectable during stage 1 (sprout period) and stage 2 (flower-bud period) (Figure 3B).

**Figure 4:**
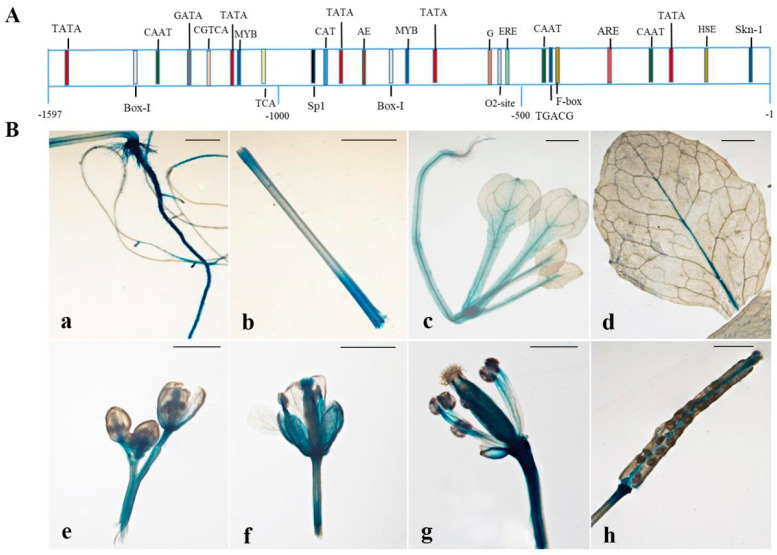
Diagram and the tissue-specific GUS activity of *CpSRG1* promoter. (**A**). A diagram of the promoter region of *CpSRG1* with potential cis-elements binding sites. (**B**). Tissue-specific GUS activity under the control of the *CpSRG1* promoter (**a**) roots, (**b**) stem, (**c**) young leaves, (**d**) senescing leaves, (**e**) young flowers, (**f**) senescing flower, (**g**) column, (**h**) silique. Bars denote 2 mm.

To further analyze the expression pattern of *CpSRG1* gene, we isolated 1597 bp of the genomic DNA sequence upstream of *CpSRG1*, including 119 bp of the 5′ untranslated region of the mRNA and the 1478 bp promoter sequence. Using the Plant CARE database, we analyzed and annotated a number of *cis*-elements in the sequence of the *CpSRG1* promoter (Figure 4A). The *cis*-acting elements of the identified *CpSRG1* promoter fragment contained basic elements (TATA-box and CAAT-box), hormone response elements (jasmonic acid response element CGTCA-motif, ethylene response element ERE and salicylic acid response element TCA), heat stress response element (HSE), endosperm-specific expression element (skn-1-motif), and light response elements (such as G-box, Sp1, I-box, and etc.). This suggests that the CpSRG1 gene may be involved in plant growth and development. Furthermore, we fused the promoter to the GUS reporter gene to generate transgenic *Arabidopsis* plants (named *CpSRG1pro::GUS*). 

Histochemical GUS staining showed that faint GUS activity was observed in the leaves, and strong GUS activity was observed in roots, stem, and flower organs. GUS staining increased from flower buds to senescing flowers. In the mature siliques, GUS staining was mainly detected in the abscission zone and upper portion of the siliques (Figure 4B). This result showed that the tissue expression pattern of the GUS gene driven by the *CpSRG1* promoter in *Arabidopsis* was consistent with the tissue-specific expression pattern of *CpSRG1* in wintersweet, with both of them being more highly expressed in senescing flowers than in other tissues. These results were consistent with the expression of *CpSRG1* in wintersweet, both of which were most abundantly expressed in senescing flowers

### 2.3. The GUS Activities of CpSRG1

**Figure 5:**
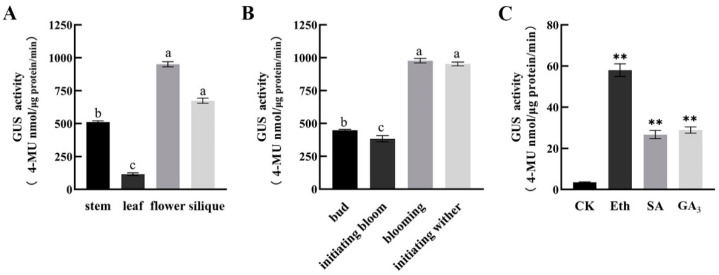
(**A**) GUS enzyme activity in different tissues. (**B**) GUS enzyme activity at different flowering stages. Mean values (±standard errors) with different superscript letters (a, b, and c) indicate significant difference (*p* < 0.05). (**C**) *CpSRG1* promoter activity in response to hormone treatments. CK, ddH_2_O; Eth, ethylene; SA, salicylic acid; GA_3_, gibberellin. Fifteen-day-old T3 homozygous *Arabidopsis* transgenic seedlings were treated with 50 µM Eth, 100 µM SA, 100 µM GA_3_ for 12 h. The plants grown under normal conditions were used as control. Data represent mean of three biological repeats ± SEM. Error bars indicate the standard error of mean. Asterisks denote statistically significant differences compared with control, ** *p* < 0.01.

To investigate the effect of exogenous hormones on *CpSRG1* expression, the GUS activity of *CpSRG1* under different hormone treatments including Eth (ethylene), SA (salicylic acid), and GA_3_ (gibberellin) were examined. These treatments were chosen according to the *cis*-elements in the promoter region of *CpSRG1*. Fifteen-day-old homozygous *CpSRG1pro::GUS* seedlings were used for the assay. The results showed that the GUS enzymatic activity significantly increased when seedlings were treated with Eth, SA, and GA_3_ (Figure 5), and suggested hormone-specific effects on the expression of *CpSRG1*. 

### 2.4. Overexpression of CpSRG1 Promoted Growth and Flowering, and Delayed Senescence in Arabidopsis 

To further investigate the function of *CpSRG1*, we transformed the *CpSRG1* gene into *Arabidopsis.* Transgenic lines were obtained through hygromycin selection, and the expression level of *CpSRG1* was confirmed by qRT-PCR. Two homozygous T3 transgenic lines (L6 and L15) with high and medium expression levels were selected for phenotypic analysis, and wild-type plants (WT) were used as a control.

**Figure 6:**
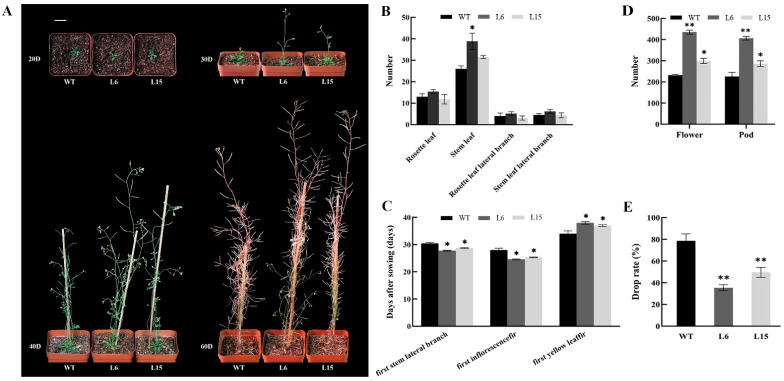
Morphological phenotype analysis of *CpSRG1* transgenic plants. (**A**) *CpSRG1* transgenic *Arabidopsis* at different stages under normal growth condition. The pictures were taken 20, 30, 40 and 60 days after sowing. Bar denotes 2 cm. (**B**) Number of leaf and branch. (**C**) The day of the first stem collateral branch, the first inflorescence and the first yellow leaf. (**D**) Number of flower and silique. (**E**) Drop rate. WT (wild-type plants, Col-0); L6 & L5, transgenic plants overexpressed *CpSRG1*. Data represent mean of three biological repeats ± SEM. Error bars indicate the standard error of mean. Asterisks denote statistically significant differences compared with control, * *p* < 0.05, ** *p* < 0.01.

**Figure 7:**
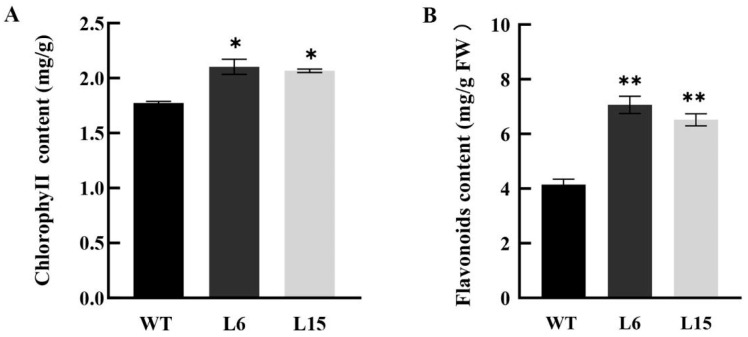
The chlorophyll and flavonoid content of leaves in *CpSRG1* transgenic and wild-type *Arabidopsis*. (**A**) chlorophyll content. (**B**) flavonoid content. WT (wild-type plants, Col-0); L6 & L5, transgenic plants overexpressed *CpSRG1*. Data represent mean of three biological repeats ± SEM. Error bars indicate the standard error of mean. Asterisks denote statistically significant differences compared with control, * *p* < 0.05, ** *p* < 0.01.

Under long day (LD) conditions, L6 and L15 exhibited faster growth, including an early flowering phenotype compared with WT plants (Figure 6A). L6 and L15 flowered at 24 and 23 days after germination, with 15.4 and 12.34 rosette leaves on average, respectively, while the WT plants flowered at 33 days after germination with 13 rosette leaves on average (Figure 6B). In addition to the early flowering phenotype, the overexpression of *CpSRG1* delayed leaf senescence in *Arabidopsis*. Compared with WT plants, L6 and L15 delayed leaf senescence by 4 and 3 days, respectively (Figure 6C), and L6 and L15 exhibited more flowers and siliques, longer internode length, and lower fruit drop rate (Figure 6D,E). Moreover, the chlorophyll and flavonoid content of leaves were increased in transgenic *Arabidopsis* (Figure 7). These data suggested that overexpression of *CpSRG1* promoted growth, flowering, and delayed senescence in *Arabidopsis*.

### 2.5. Overexpression of CpSRG1 Improved the Utilization of Nitrogen in Arabidopsis 

**Figure 8:**
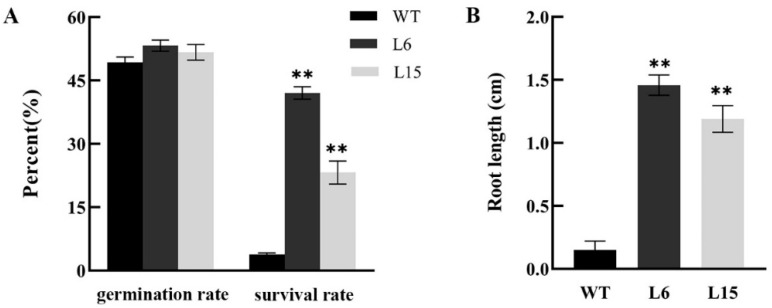
Low nitrogen tolerance analysis of *CpSRG1* transgenic and wild-type Arabidopsis. (**A**) Germination rate and survival rate under low nitrogen treatment. (**B**) Root length. WT (wild-type plants, Col-0); L6 & L5, transgenic plants overexpressed *CpSRG1*. Data represent mean of three biological repeats ± SEM. Error bars indicate the standard error of mean. Asterisks denote statistically significant differences compared with control, ** *p* < 0.01.

The previous results showed that overexpression of *CpSRG1* promoted growth and flowering (Figure 6). We speculated that the *CpSRG1* gene may promote the synthesis of assimilates in *Arabidopsis*, thereby improving growth and flowering. Therefore, we analyzed the response of *CpSRG1* transgenic *Arabidopsis* to low nitrogen stress, and the results showed that there was no significant difference in the germination rate of the transgenic lines as compared with the wild-type. However, the survival rates of the transgenic lines were significantly higher, and the root lengths of the transgenic lines were significantly longer than those of the wild-type plants under low nitrogen stress (Figure 8). 

**Figure 9:**
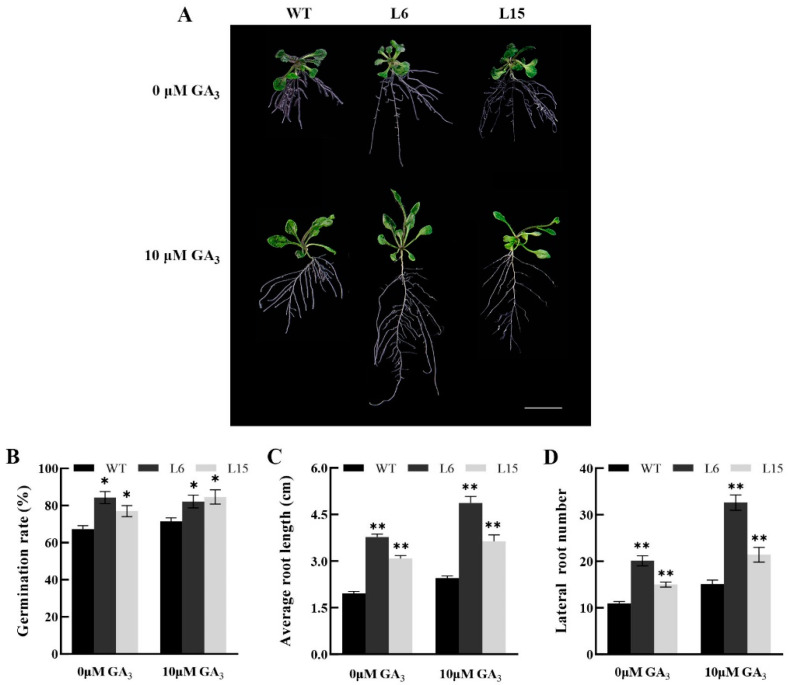
Effects comparison of GA_3_ treatment on *CpSRG1* transgenic and wild-type *Arabidopsis*. (**A**) The phenotype of fifteen-day-old plants under GA_3_ treatment. Bar denotes 1 cm. (**B**) Germination rate. (**C**) Average root length. (**D**) lateral root number. WT (wild-type plants, Col-0); L6 & L5, transgenic plants overexpressed *CpSRG1*. Data represent mean of three biological repeats ± SEM. Error bars indicate the standard error of mean. Asterisks denote statistically significant differences compared with control, * *p* < 0.05, ** *p* < 0.01.

To further analyze the function of *CpSRG1*, we treated transgenic and wild-type *Arabidopsis* with 10 µM GA_3_ (gibberellin). The results showed that the root length and lateral root number were promoted in the transgenic and wild-type *Arabidopsis* under GA_3_ treatment. Additionally, the survival rates of the transgenic lines were significantly higher, and the root lengths of the transgenic lines were significantly longer than those of the wild-type plants under GA_3_ treatment (Figure 9). These results indicated that the seed germination and growth may be promoted in *CpSRG1* transgenic *Arabidopsis* through the GA pathway.

## 3. Discussion

The 2-oxoglutarate-dependent dioxygenase (2OGD) superfamily is a large enzyme family that contains many proteins involved in hormone biosynthesis and metabolism [25]. The 2OGD superfamily can be divided into the DOXA, DOXB, and DOXC subfamilies based on the amino acid sequence [26]. DOXAs are plant homologues of the *Escherichia coli* DNA repair enzyme AlkB, which are mainly involved in the repair of oxidative demethylation of nucleic acids and histones damaged by alkylation. 

Recently, it was discovered that there are two *Arabidopsis* DOXA subfamilies, the close homologues ALKBH9B and ALKBH10B, which can demethylate m6A in RNA [27,28]. DOXBs are mainly involved in the proline hydroxylation reaction catalyzed by the P4H enzyme (prolyl 4-hydroxylase) in the process of cell wall protein synthesis. Proper cell wall structure and root tip growth require the hydroxylation of extension proteins, and P4H2, P4H5, and P4H13 are involved in this process in *Arabidopsis* [29]. DOXC proteins play important roles in plant growth and development, and are involved in the metabolism of different plant phytochemicals, including plant hormones and flavonoids [26]. Biosynthesis and metabolism of phytohormones such as Eth (ethylene) [30], JA (jasmonic acid) [31], GAs (gibberellins) [12], SA (salicylic acid) [13], IAA (indole-3-acetic acid) [32], and SLs (strigolactones) [33] are all related to DOXC proteins. To date, nine hormone biosynthesis and metabolism-related protein families have been identified in the 2OGD family, including gibberellin 20 oxidase (GA20oxs), gibberellin 3 oxidase (GA3oxs), gibberellin 2 oxidase (GA2oxs), DAOs, ACO, JOX, DMR6s, DLO, and LBO [11]. Moreover, the number of DOXC has increased from bryophytes to angiosperms, which is consistent with the increasing complexity of specialized metabolism [26]. Here, we identified and characterized a senescence-related gene, *CpSRG1*, from wintersweet. After multiple sequence alignment, it was determined that the DIOX_N motif and 2OD_FeII_Oxy motif in the sequence of CpSRG1 were in accord with the characteristics of the DOXC subfamily (Figure 1). Phylogenetic analysis showed that CpSRG1 was closely related with OsSRG1 and AtaSRG1 (Figure 2).

The expression patterns of a gene can reflect its function to some extent. In different plants, the gene expression pattern of the 2-OGD family members is different. For example, in sweet cherry (*Prunus avium* L.), *PaFLS* exhibits the highest expression in the flowers as compared to that in the fruits, pholems, and leaves, which may be related to the participation of flavonols in pollen germination and pollination [34]. *JcGA20oxs* from *Jatropha curcas* was abundantly expressed in stems, but scarcely in leaves [35]. For the same *Cv20oxs* from watermelon, the gene was strongly expressed in the developing seeds and integument tissues, but weakly in inner seed tissues [36]. *GmDAO1* exhibited the lowest expression in leaves and the highest expression in roots because it may be involved in the metabolic pathway of auxin [37]. In this study, the tissue-specific expression pattern showed that *CpSRG1* was expressed in almost all the tissues of wintersweet and was highly expressed in the inner petals and stamens (Figure 3A). 

In addition, it was reported that differential gene expression occurred for the 2-OGD family members during flower development stages. For example, *LrF3H* was expressed during the entire flower development process, and the expression level gradually increased as the coloring of the petals deepened from the initial bud stage to the blooming stage. Similarly, the expression level was the highest in the blooming stage, and then, the expression level decreased as the flowers of *Lycoris radiata* wilted [38]. In grape hyacinth, expression analysis revealed significant differences during five different floral developmental stages in three different cultivars. *MaFLS1* had the highest expression at stage 2 in ‘White Beauty’, stage 3 in ‘Pink Surprise’ and stage 4 in ‘Muscari armeniacum’ [39]. During wintersweet flower development, obvious expression of *CpSRG1* was detected from stage 3 (petal-display period) to stage 5 (bloom period) and reached the highest expression at stage 6 (withering period) (Figure 3B). These results indicate that *CpSRG1* may play roles in flower development and senescence in wintersweet. Corresponding with the expression patterns of *CpSRG1*, the *cis*-elements that were considered to be involved in plant growth and development, such as Sp1 and CAT-box, and which respond to ethylene (ERE) and SA (TCA-element), were found in the promoter sequence of *CpSRG1* (Figure 4A). In transgenic *CpSRG1pro::GUS Arabidopsis*, GUS activity was highly detected in roots, stems, and flower organs, and GUS staining was increased when the flower was senescing, which is consistent with the tissue-specific expression pattern of *CpSRG1* in wintersweet. Moreover, the GUS enzymatic activity was significantly increased when transgenic *CpSRG1pro::GUS* seedlings were treated with ethylene, SA, or GA_3_, which suggested that the expression of *CpSRG1* was influenced by different hormones (Figure 5). 

It has been reported that SRG proteins are involved in plant developmental and physiological processes. In the current study, the overexpression of *CpSRG1* promoted growth and flowering, and delayed senescence in transgenic *Arabidopsis* (Figure 6). Additionally, transgenic *CpSRG1* plants exhibited higher chlorophyll and flavonoid content than wild-type plants (Figure 7). According to previous studies, in the development of tapetum, 2-OGD family proteins were downregulated in male sterile lines in *Brassica oleracea,* thereby influencing flower development [40]. Proteins of the DOXB clade, the *icu11* mutants, cause hyponastic leaves and early flowering in *Arabidopsis.* In addition, overexpressing lines had a three-day longer stay-green period [41]. 

Plant growth and senescence are also influenced by different plant hormones. For example, ethylene and salicylic acid coordinate to promote leaf senescence [42]. Gibberellins (GA) delay leaf senescence, such as that of rose [43] and *Paris polyphylla* [44], but it has also been reported that exogenous GA_3_ treatment promoted the senescence of *Arabidopsis* rosette leaves [45]. *BrTCP7* is related to leaf senescence promoted by methyl jasmonic acid (MeJA) by activating JA biosynthesis and chlorophyll catabolism [46]. In the current study, overexpression of *CpSRG1* resulted in a higher survival rate and significantly longer root length in transgenic lines than in the wild-type plants under low nitrogen stress (Figure 8). Moreover, the survival rates of the transgenic lines were significantly higher, and the root lengths of the transgenic lines were also significantly longer than the wild-type plants under GA_3_ treatment (Figure 9). The *CpSRG1* gene may promote the synthesis of plant assimilates through the GA pathway, thereby improving growth and flowering, and delaying senescence in transgenic *Arabidopsis*. 

Thus far, there has been little research on the *SRG1* gene, and the function of *SRG1* is still not very clear. There is currently no reported research on the *SRG1* gene of wintersweet (*Chimonanthus praecox*). Hence, our study has laid a satisfactory foundation for further analysis of senescence-related genes in wintersweet. Our data provide clues regarding the roles that the *SRG1* gene may play in woody plants through its functional characterization. Moreover, these finding also enrich our knowledge of the 2OG-Fe(II) dioxygenase superfamily, which plays a variety of important roles in plants and has not yet been well studied. 

## 4. Materials and Methods

### 4.1. Plant Materials and Growth Conditions

Wintersweet flowers were collected from Southwest University. To ascertain the expression of the *CpSRG1* gene in different flower stages, we collected flowers at six stages, including stage 1 (sprout period), stage 2 (flower-bud period), stage 3 (display-petal period), stage 4 (initiation of bloom period), stage 5 (bloom period), and stage 6 (wither period) [47]. For the tissue-specific expression of *CpSRG1*, the root, stem, leaves, petals, stamens, and pistils were collected, with three biological replicates for each sample. 

For plant transformation, the seeds of *A. thaliana* ecotype Columbia were sown on Murashige and Skoog (MS) solid medium containing 3% (*w*/*v*) sucrose and 0.7% (*w*/*v*) agar, stratified for three days at 4 °C, and then transferred to a culture room under a 16 h light (22 °C)/8 h dark (20 °C) cycle with 70% relative humidity and 20,000 lux lighting conditions. Twelve days later, seedlings were transplanted into a soil mixture containing substrate and vermiculite (1:1, *v*/*v*) and were grown in a culture room under a 16 h light (22 °C)/8 h dark (20 °C) cycle with 70% relative humidity.

### 4.2. Isolation of CpSRG1 and Its Promoter

Total RNA was extracted using an RNAprep Pure kit (Tiangen Biotech, Beijing, China) and then used to synthesize the first-strand cDNA with a PrimerScript RT reagent Kit with gDNA Eraser (TaKaRa, Dalian, China). A randomly expressed sequence tag (EST) sequence was identified from the wintersweet cDNA library [47], and the full-length gene cDNA was amplified using specific primers (Table S1). Then, the amplified fragment was inserted into the pMD19-T Easy vector (TaKaRa) and sequenced by the Huada Gene Technology Co., Ltd., Shanghai, China. This cDNA comprised 1489 bp, and the deduced amino acid sequence showed a high similarity to the SRG protein. We named this gene *CpSRG1*. Multiple sequence alignments were carried out using the DNAMAN 8.0 program. A phylogenetic tree for CpSRG1 and SRG proteins from other species was constructed using Molecular Evolutionary Genetics Analysis version 7.0 (http://www.megasoftware.net/ (accessed on 22 November 2019)) based on the neighbor joining (NJ) method. 

The 5′-upstream sequence of *CpSRG1* was isolated using hiTAIL-PCR [48]. The specific nested primers and degenerate primers were designed based on the 5′-end sequence of *CpSRG1* (Table S1). A sequence 1597 bp in length was obtained by PCR. A search was conducted to find the putative cis-acting regulatory elements using the PlantCARE program (http://bioinformatics.psb.ugent.be/webtools/plantcare/html/ (accessed on 10 March 2020)).

### 4.3. Plasmid Constructs

The open reading frame (ORF) of *CpSRG1* was amplified by the primers p-*CpSRG1*-F and p-*CpSRG1*-R (Table S1), then cloned into the plant binary vector pCAMBIA 2301G. The recombinant plasmid 35S:*CpSRG1-GUS* (*β-glucuronidase*) was sequenced for confirmation. The *CpSRG1* promoter region was amplified by the primers *CpSRG1*-P-F and *CpSRG1*-P-R (Table S1), and then ligated into the pBI121 vector to construct the plasmid *CpSRG1pro::GUS*. The recombinant plasmids 35S:*CpSRG1-GUS* and *CpSRG1pro::GUS* were separately introduced into *Agrobacterium tumefaciens* strain GV3101. 

### 4.4. Plant Treatment

To understand the effects of different treatments on the *GUS* gene driven by the *CpSRG1* promoter in *Arabidopsis*, fifteen-day-old homozygous *Arabidopsis* T3 seedlings were treated with 50 µM Eth (ethylene), 100 µM SA (salicylic acid) and 100 µM GA_3_ (gibberellin) for 12 h, respectively [24,49,50]. Seedlings grown under normal conditions were used as controls. These samples were then used for GUS activity analysis. Each experiment contained three biological replicates. 

To analyze the response of *CpSRG1* transgenic *Arabidopsis* to low nitrogen stress and GA_3_ treatment, the seeds of wild-type (WT) and T3 transgenic *Arabidopsis* were sown on MS medium or MS medium supplemented with 4.7 mM nitrogen or 10 µM GA_3_, respectively. Then, the seedlings were cultivated in a light incubator after three days of vernalization under a 16 h light (22 °C)/8 h dark (20 °C) cycle at 70% relative humidity. 

### 4.5. Quantitative Real Time-PCR Analysis

qRT-PCR was performed on a Bio-Rad CFX96 Real-time System machine using the Sofast EvaGreen Supermix (Bio-Rad, Hercules, CA, USA). The PCR conditions were as follows: 95 °C for 30 s, followed by 40 cycles of 95 °C for 5 s, 60 °C for 5 s and 72 °C for 5 s, and a melt cycle from 65 °C to 95 °C. The primers used for real-time PCR (Table S1) were designed by Primer Premier 5.0 software. *CpActin* and *CpTubulin* were used as the internal reference for the gene expression analysis of wintersweet [47]. Bio-Rad Manager^TM^ Software (Version 1.1) was used to analyze real-time PCR data. There were three biological replicates for each experiment and three technical replicates for each sample.

### 4.6. Arabidopsis Transformation

For GUS histological assays, the recombinant plasmid *CpSRG1pro::GUS* was introduced into *A*. *tumefaciens* strain GV3101 and then transformed into *Arabidopsis* via the floral dipping transformation method [51]. T0 seeds were sown on MS medium containing 50 mg/L kanamycin, and the positive seedlings were subsequently used in experiments. *CpSRG1* overexpression plants were generated, as described above, and the positive seedlings were identified by growing on MS media containing 25 mg/L hygromycin. The positive seedlings were grown in a culture room under a 16 h light (22 °C)/8 h dark (20 °C) cycle with 70% relative humidity and 20,000 lux lighting conditions. 

For GUS histological assays, the recombinant plasmid *CpSRG1pro::GUS* was introduced into *A*. *tumefaciens* strain GV3101 and then transformed into *Arabidopsis* via the floral dipping transformation method [51]. T0 seeds were sown on MS medium containing 50 mg/L kanamycin, and the positive seedlings were subsequently used in experiments. *CpSRG1* overexpression plants were generated, as described above, and the positive seedlings were identified by growing on MS media containing 25 mg/L hygromycin. The positive seedlings were grown in a culture room under a 16 h light (22 °C)/8 h dark (20 °C) cycle with 70% relative humidity and 20,000 lux lighting conditions. 

### 4.7. GUS Histochemical and GUS Activity Assays

Histochemical assay of GUS and enzymatic activity assay were performed according to Jefferson et al. [52]. The plants were incubated overnight at 37 °C in 2 mM X-Gluc (5-bromo-4-chloro-3-indolyl b-D-glucuronide) with a phosphate buffer (pH 7.0) containing 10 mM ethylenediaminetetraacetic acid (EDTA), 0.5 mM potassium ferricyanide, 0.5 mM potassium ferrocyanide, and 0.1% *v/v* Triton X-100. By using 70% ethanol after X-Gluc staining, chlorophyll was completely removed from the plants, which were then photographed using a stereoscopic microscope (Nikon, Tokyo, Japan). 

The procedure for the GUS fluorometric assay was performed according to Niu et al. [53]. GUS protein can react with the substrate 4-methylumbelliferyl-β-d-glucuronic acid (MUG, Sangon, Shanghai, China) to generate 4-MU (4-methylumbelliferone). GUS enzymatic activity is expressed as pmol 4-MU produced per milligram protein per minute. GUS activity was measured using a Varioskan Flash Spectral Scanning Multimode Reader (Thermo Fisher, Waltham, MA, USA) with 365 nm excitation and 455 nm emission. 

### 4.8. Phenotypic Analysis

For phenotypic observation, 5–10 strains from each transgenic line and WT were selected. The phenotypes were observed from the seedling stage to the senescence stage, and statistical data was recorded. The flowering time was measured as described by Zhang et al. [54]. The total rosette leaf number was counted when the first visible flower appeared. 

### 4.9. Analysis of Chlorophyll and Flavonoids Content 

The chlorophyll content was extracted by ethanol and acetone extraction (1:1) [55], and the chlorophyll levels were quantified by the absorbance value, which was measured with a Varioskan Flash multimode microplate reader (Thermo Fisher Scientific, Waltham, MA, USA) at 663 nm and 646 nm. The flavonoids of rosette leaf were extracted followed by Routaboul et al. [56] and measured, as described by Routaboul et al. [57].

### 4.10. Statistical Analysis

Statistical analysis was conducted using SPSS software (version 22.0; IBM Corporation, Armonk, NY, USA). The significance of differences was analyzed by Student’s *t*-test, and significance was indicated with *p*-value < 0.05, and very significant with *p*-value < 0.01. 

## 5. Conclusions

In summary, we identified a senescence-associated gene *CpSRG1*, belonging to the 2OG-Fe(II) dioxygenase superfamily from wintersweet (*Chimonanthus praecox*). In addition, the tissue expression pattern of the GUS gene driven by the *CpSRG1* promoter in *Arabidopsis* was consistent with the tissue-specific expression pattern of *CpSRG1* in wintersweet, both of which were most abundantly expressed in senescing flowers. It was also found that exogenous Eth (ethylene), SA (salicylic acid), and GA_3_ (gibberellin). could enhance the activity of *CpSRG1* promoter. Moreover, heterologous overexpression of *CpSRG1* in *Arabidopsis* promoted growth and flowering, and delayed senescence. Under low nitrogen stress and GA treatment, the survival rates were significantly higher and the root lengths were significantly longer in the transgenic lines than in the wild-type plants. This indicated that the *CpSRG1* gene may promote the synthesis of assimilates in plants through the GA pathway, thereby improving growth and flowering, and delaying senescence in transgenic *Arabidopsis*.

## Data Availability

Not applicable.

## Supplementary Materials

The following supporting information can be downloaded at: https://www.mdpi.com/article/10.3390/ijms232213971/s1.
