# Supplementary material for: Overexpression of a Senescence-Related Gene CpSRG1 from Wintersweet (Chimonanthus praecox) Promoted Growth and Flowering, and Delayed Senescence in Transgenic Arabidopsis
Source: Int J Mol Sci. 2022 Nov 12;23(22):13971. doi: 10.3390/ijms232213971 (PMC9696086; doi:10.3390/ijms232213971)
Supplement: Supplementary file 1 [file ijms-23-13971-s001.zip › ijms-1987481-supplementary.pdf]

Table S1. List of primers

Usage	Primer name	Primer sequence (5'−3')
cloning of *CpSRG1* and its promoter	CpSRG1-F	CCGAGCCGCGTGAGCTGCTAC
	CpSRG1-R	CCGCATGCATAAGCTTGCTCGAG
	LAD1	ACGATGGACTCCAGAGCGGCCGCVNVNNNGGAA
	LAD2	ACGATGGACTCCAGAGCGGCCGCBNBNNNGGTT
	LAD3	ACGATGGACTCCAGAGCGGCCGCVVNVNNNCCAC
	LAD4	ACGATGGACTCCAGAGCGGCCGCBDNBNNNCCAA
	LAD5	ACGATGGACTCCAGAGCGGCCGCBDNBNNNCGGT
	AC0	GGACGATGGACTCCAG
	CpSRG1-RB0	CAAATCCCTCAAGATCGTCTGCCTG
	CpSRG1-RB1	ACGATGGACTCCAGTCCGGCCAGGGATACATCAATGGC TGGTGGGTC
	AC1	ACGATGGACTCCAGAG
	CpSRG1-RB2	GCGAGATGGGACGGTGTTCTTATGC
Plasmid constructs	p-CpSRG1-F	CGGGATCCATGGTGAGCCTTGGAACTTCAGTTC
	p-CpSRG1-R	CGAGCTCTTAATCAGTAGCAACAGCAGCACTG
	CpSRG1-P-F	CGCAAGCTTGTCAATTCTCTGAAGAACAAGTACC
	CpSRG1-P-R	CCCGGATCCTTCCTCTGTTTCTTCAATTTGCT
Quantitative Real Time-PCR	qCpSRG1-F	TAAGAACACCGTCCCATCTCGCTAC
	qCpSRG1-R	GAGTGCAGTCTCTCCAATTCATCAG
	qActin-F	GTTATGGTTGGGATGGGACAGAAAG
	qActin-R	GGGCTTCAGTAAGGAAACAGGA
	qTublin-F	TAGTGACAAGACAGTAGGTGGAGGT
	qTublin -F	GTAGGTTCCAGTCCTCACTTCATC

Note: The underlined bases in the primers are the restriction sites.

N stands for A/G/C/T; V stands for G/A/C; B stands for G/T/C; D stands for G/A/T
